# Genomic analysis of Salehabad virus obtained from the field after a 64-year silence

**DOI:** 10.1007/s00705-026-06701-6

**Published:** 2026-07-30

**Authors:** Daisuke Kobayashi, Kardelen Yetismis, Suha Kenan Arserim, Metin Pekagirbas, Kentaro Itokawa, Haruhiko Isawa, Seray Toz, Yusuf Ozbel, Chizu Sanjoba, Shinji Kasai

**Affiliations:** 1 Department of Medical Entomology, National Institute of Infectious Diseases, Institute for Health Security, Tokyo, Japan; 2 Department of Parasitology, Faculty of Medicine, Ege University, Izmir, Türkiye; 3 Vocational School of Health Sciences, Manisa Celal Bayar University, Manisa, Türkiye; 4 Department of Parasitology, Faculty of Veterinary Medicine, Aydin Adnan Menderes University, Aydin, Türkiye; 5 Department of Animal Resource Sciences, Graduate School of Agricultural and Life Sciences, The University of Tokyo, Tokyo, Japan

## Abstract

This study reports the first re-isolation of a wild-type Salehabad virus (SALV) since 1959, from a pool of male sand flies collected in Türkiye in August 2023. The isolate and the 1959 prototype strain shared 86–92% nucleotide identity across the four coding sequences but 95–99% amino acid identity; for the L protein, 12% nucleotide divergence corresponded to 1% amino acid divergence. Because male sand flies do not blood-feed, this isolation indicates transmission between sand flies by a route other than blood-feeding. These findings highlight the cryptic circulation of SALV and underscore the importance of monitoring historically overlooked phleboviruses.

**Supplementary Information:**

The online version contains supplementary material available at 10.1007/s00705-026-06701-6.

Sandfly-borne phleboviruses are distributed globally, including in Africa and the Americas [1]. However, they pose major public health threats primarily in regions spanning from the Mediterranean Basin to the Middle East and Central Asia, where they cause diseases such as pappataci fever and Toscana virus (TOSV)-induced encephalitis [2]. Traditionally, medical emphasis has been limited to a subset of pathogenic viruses, such as the Sandfly fever Sicilian virus [2]. Recent advances in diagnostic technologies have suggested that a diverse array of novel viruses may be involved in human disease [3]. The Salehabad virus (SALV) species complex was previously considered to have low pathogenicity and negligible public health significance [4]. Recently, however, its association with clinical diseases has been re-evaluated, as related viruses (e.g., Adria virus) have been detected in cases of pediatric aseptic meningitis [4, 5]. Since the isolation of the prototype strain, I-81, in Iran in 1959, no subsequent wild-type strains have been reported [6, 7]. Consequently, the maintenance mechanisms and genetic dynamics of SALV remain unclear.

To elucidate the potential circulation dynamics of these viruses in the Aegean coastal region of Türkiye, an active endemic area for TOSV and various novel phleboviruses [2, 8], an extensive field survey of sand flies was conducted in August 2023. Adult sand flies were collected using Centers for Disease Control and Prevention (CDC) miniature light traps (John W. Hock Company, Gainesville, FL, USA) placed around livestock shelters. Sand flies were sexed on the basis of external morphology and then pooled by sex and collection site. Virus isolation was performed using Vero cells following the methodology described previously [9]. A clear cytopathic effect (CPE) was observed as early as two days post-inoculation in cells inoculated with a pool consisting of 20 male sand flies (pool ID: 23SF8) collected from Didim (37.417669, 27.375478), Aydin Province, Türkiye. No CPE was observed in the other sand fly samples subjected to isolation [a total of 15 pools, including eight female pools (derived from 358 individuals) and seven male pools (derived from 149 individuals)]. When the culture supernatant was inoculated into freshly-prepared Vero cells, an identical CPE was observed. The recovered culture supernatant was re-inoculated into fresh Vero cells. The cells were then fixed (glutaraldehyde and paraformaldehyde in phosphate buffer, followed by post-fixation with osmium tetroxide), dehydrated, and embedded in epoxy resin. Transmission electron microscopy (TEM) of the ultrathin sections was conducted by Tokai Electron Microscopy, Inc. (Aichi, Japan). TEM analysis revealed clusters of spherical particles, approximately 80 nm in diameter, characteristic of phleboviruses, in the cytoplasm and extracellular regions (Fig. 1).

**Fig. 1:**
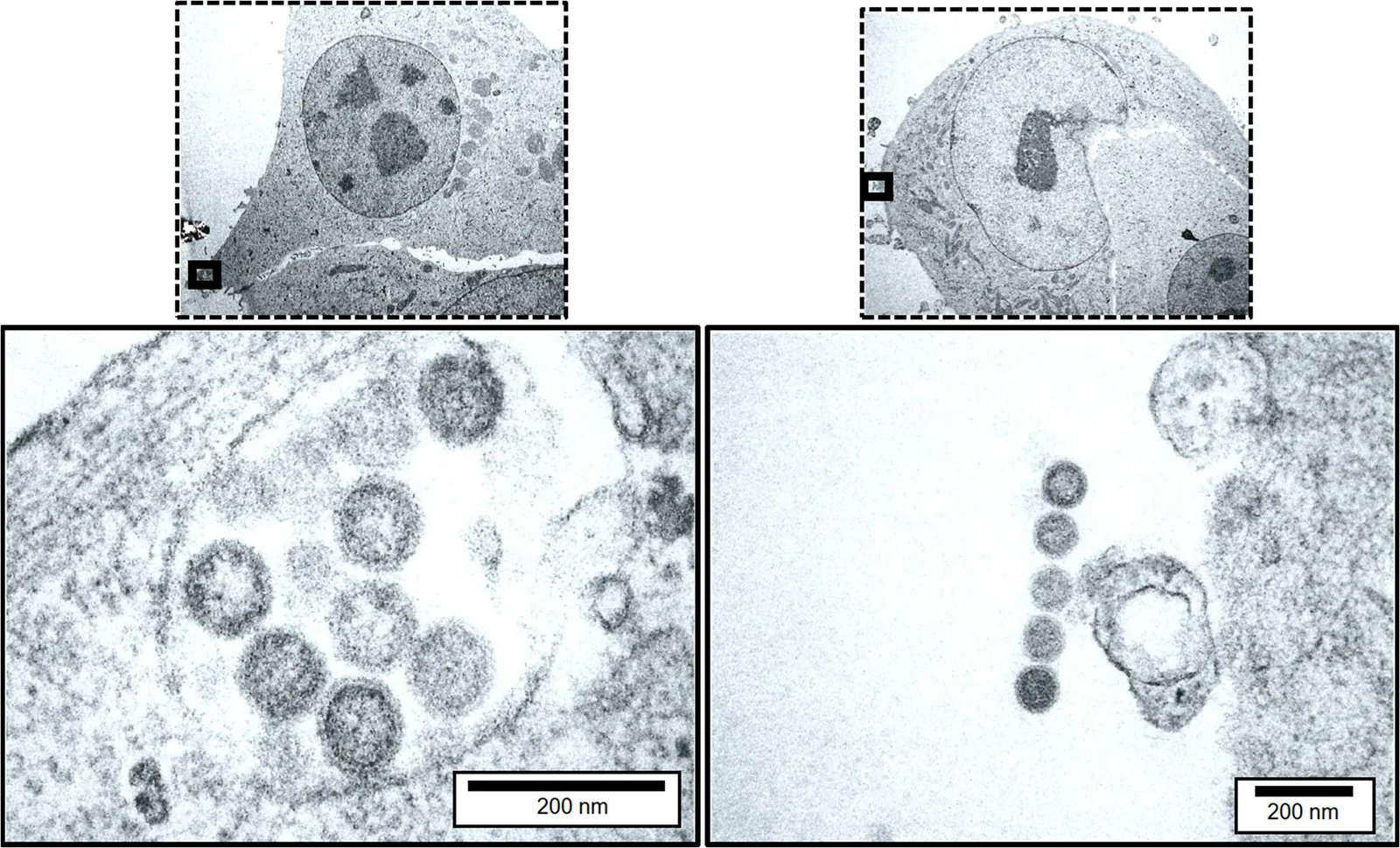
Transmission electron micrographs of Vero cells inoculated with pool no. ID 23SF8. Ultrathin sections of Vero cells were fixed and observed one day post-inoculation. The upper panels (dashed boxes) provide an overview of the infected cells containing viral particles. The lower panels show magnified views of the areas enclosed by the solid black squares in the upper panels. The scale bars in the lower panels represent 200 nm

Subsequently, comprehensive analysis of the culture supernatant of the third Vero cell passage was conducted using next-generation sequencing (NGS), as described previously [10]. This analysis detected sequences related to those of the SALV prototype strain. Terminal sequences were determined using the rapid amplification of cDNA ends (RACE) method [11]. This analysis enabled the successful determination of full-length viral genome sequences (L segment, 6,402 nt; M segment, 4,224 nt; S segment, 1,765 nt) that possessed terminal sequences common to phleboviruses. These sequences were deposited in the International Nucleotide Sequence Database Collaboration (DDBJ/ENA/GenBank) with accession numbers LC938656 (L segment), LC938657 (M segment), and LC938658 (S segment). Each segment exhibited a genomic structure typical of the genus, *Phleboviru*s (a single large open reading frame on the L and M segments and an ambisense genomic structure on the S segment) [12]. Nucleotide and amino acid sequences were compared with those of known phleboviruses by pairwise alignment based on the Lipman–Pearson method [13] using GENETYX version 13 (GENETYX Corporation, Tokyo, Japan). The highest sequence homology was with the prototype SALV strain (I-81). The amino acid sequence identities were 99% for the L protein; 95% for the glycoprotein precursor (GPC) including the nonstructural protein M (NSm); 97% for the nucleoprotein (N); and 98% for the nonstructural protein S (NSs). The corresponding nucleotide sequence identities between the coding sequences of 23SF8 and I-81 were substantially lower: 88% (L), 86% (GPC), 90% (N) and 92% (NSs) (Table 1).

**Table 1:** Nucleotide and amino acid sequence identities between isolate 23SF8 and related viruses

		Sequence identity vs. 23SF8 (%)							
		L		GPC		*N*		NSs	
Virus name	sitrain	nt^#^	aa^##^	nt	aa	nt	aa	nt	aa
Salehabad virus	I-81	88	99	86	95	90	97	92*	98*
Zaba virus	C48	80	95	65	67	82	95	80*	84*
Medjerda Valley virus	T131	77	89	68	71	77	87	68	71
Arbia virus	ISS PHL18	76	89	63	62	77	87	68	70
Alcube virus	S20	76	89	63	63	80	89	65	69
Shable virus	SP109-KE-2019	75	88	69	74	75	85	66	69
Adana virus	195	75	85	65	66	73	78	68	73
Bregalaka virus	M31	74	85	70	76	73	79	69*	74*
Grapi virus	Kosovo.SP59	74*	85*	70	76	73	79	68*	74*
Ponticelli I virus	194,246/2013	74*	85*	68	74	71*	78*	69	74
Ponticelli II virus	238134-4/2016	74*	85*	69*	76*	72	78	69	75
Ponticelli III virus	195684-2/2016	74*	85*	68	74	71	79	68	74
Odrenisrou virus	ArA1131/80	NT**	64	NT	41	NT	48	NT	39
Arumowot virus	Ar 1286-64	NT	63	NT	39	NT	46	NT	38
Salanga virus	AnB 904a	NT	59	NT	42	NT	55	NT	24
Sandfly fever Sicilian virus	Izmir 19	NT	55	NT	36	NT	43	NT	24

^#^nucleotides

^##^amino acids

*partial coding sequence

** not tested

The viral sequences used in this study are shown in Supplementary Table 1

Phylogenetic analysis based on amino acid sequences revealed that this isolate formed a robust monophyletic clade with the SALV prototype strain for all viral proteins (Fig. 2). Since this satisfied the current species demarcation criteria for the genus *Phlebovirus* set by the International Committee on Taxonomy of Viruses (ICTV) (less than a 5% difference in the L protein amino acid sequence) [14], the virus was identified as SALV. This represents the first global re-isolation of a wild-type SALV strain since its initial discovery in 1959.

**Fig. 2:**
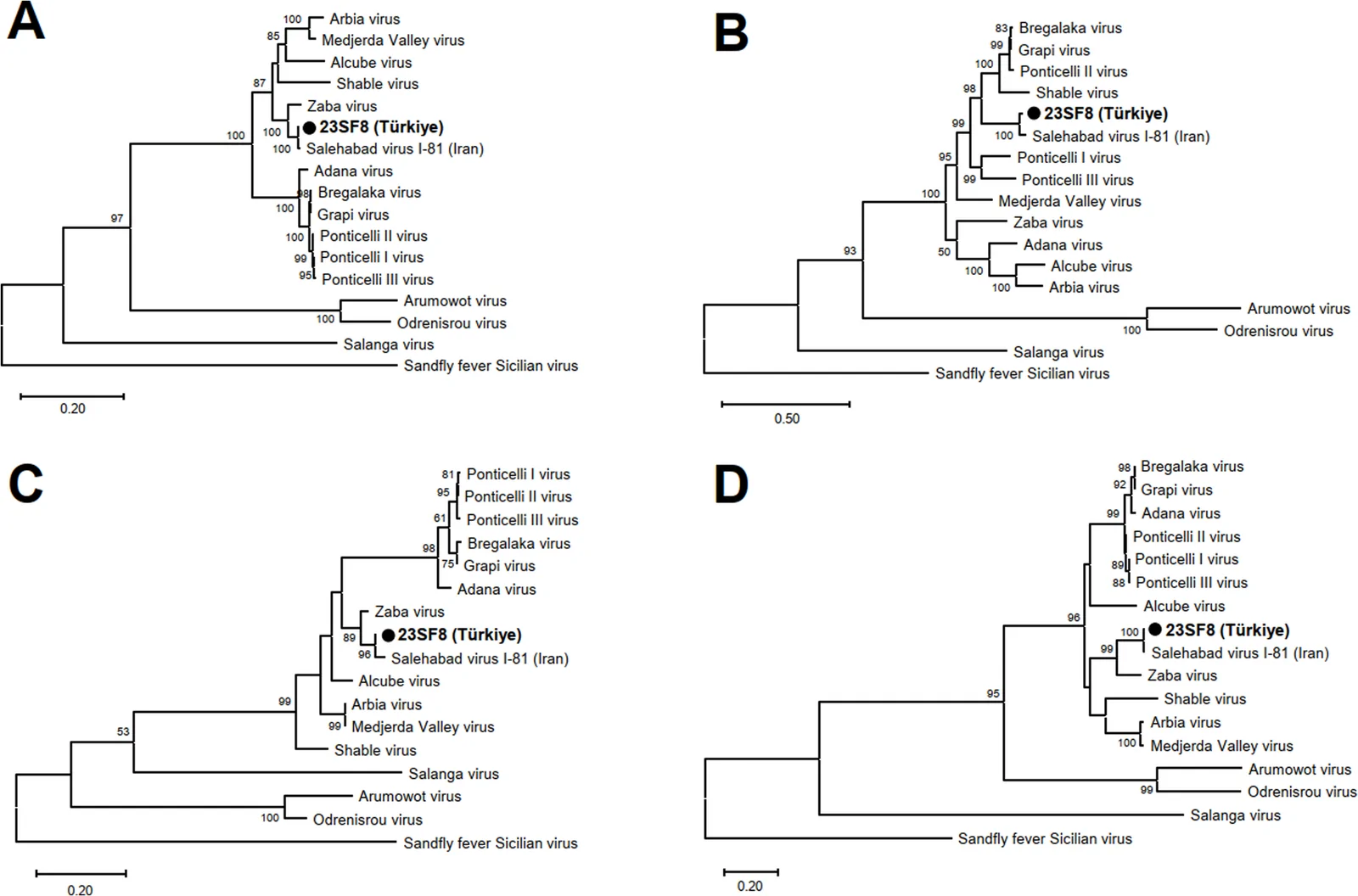
Maximum-likelihood (ML) phylogenetic trees based on the amino acid sequences of viral proteins. Trees are shown for the (**A**) L protein; (**B**) glycoprotein precursor (GPC) containing the nonstructural protein M; (**C**) nucleoprotein (N); and (**D**) nonstructural protein S (NSs). The virus isolated in this study is indicated in bold and marked with a solid circle. Amino acid sequences were aligned using the L-INS-i strategy of the MAFFT online service [15], and divergent or ambiguously aligned regions were removed using Gblocks version 0.91b [16]. Trees were constructed using the ML method in MEGA 12 [17] with the best-fit amino acid substitution model selected for each alignment (L, LG + G+I + F; GPC, LG + G; N, LG + G; NSs, LG + G). Numbers at the nodes indicate bootstrap values (1,000 replicates) as percentages; only values ≥ 50% are shown. The trees were rooted using sandfly fever Sicilian virus as an outgroup. Scale bars indicate the number of amino acid substitutions per site. The viral sequences used in this study are listed in Supplementary Table 1

To further determine the species composition of the SALV-positive sand fly pool, we performed metabarcoding to identify the species. Total DNA was extracted from the homogenate of the SALV-positive pool using NucleoSpin Blood (Takara Bio, Shiga, Japan). Species composition was assessed using a previously established sand fly metabarcoding protocol [18]. Briefly, the 18 S ribosomal DNA (rDNA) region was PCR-amplified, and the resulting amplicon was analyzed by NGS as described above. The generated sequence reads were subsequently analyzed using the CLC Genomics Workbench 21 (QIAGEN, Limburg, Nederland). The analysis revealed that approximately 99% of the total reads (997,749) shared > 99% sequence identity with the 18 S rDNA of *Phlebotomus tobbi*. The remaining 1% of the reads (19,085) exhibited a 100% sequence identity with *P. simici*. These findings demonstrate that the SALV-positive pool was predominantly composed of *P. tobbi*, with minor inclusion of *P. simici*.

The findings of this study provide insights into the evolutionary dynamics of SALV. Sixty-four years elapsed between the initial discovery of SALV in 1959 and the present re-isolation. The prototype I-81 and isolate 23SF8 differ by 8–14% at the nucleotide level across their coding sequences, whereas their proteins are 95–99% identical; for the L protein, 12% nucleotide divergence corresponds to only 1% amino acid divergence (Table 1). Among the viruses examined here, 23SF8 and I-81 are the most closely related pair; the next closest, Zaba virus, shares 80% nucleotide and 95% amino acid identity in the L protein (Table 1). The historical absence of SALV isolation reports is unlikely to reflect the disappearance of the virus. Field surveys of sand flies in Iran, where SALV was originally isolated, recovered other phleboviruses in 2009–2011 [19] and in 2019–2020 [20] but not SALV, and in Türkiye several phleboviruses, including Adana virus of the SALV complex, have been detected or isolated [2, 4, 8, 21], yet SALV itself has not been reported since 1959.

Male phlebotomines do not blood-feed. The isolation of an infectious virus from a pool of male sand fly specimens therefore indicates that SALV is transmitted between sand flies by a route other than blood-feeding. Transovarial transmission has been reported for sand fly-borne phleboviruses, supported by field isolations from non blood-feeding males and laboratory experiments on other phleboviruses [22, 23], and venereal transmission between sandflies has also been described in this group [21]. The present data derive from a single isolation and cannot determine by which route these males became infected. The possibility that SALV circulates within sand fly populations with little dependence on vertebrates carries the inherent risk that previously overlooked potential virus could emerge in response to environmental changes.

This study has several limitations. The virus was recovered from a single pool, and the sequences reported here derive from virus passaged three times in Vero cells, so culture-associated changes cannot be excluded. Because I-81 and 23SF8 were obtained from different countries, their divergence reflects geographic separation in addition to the elapsed interval, and the present data do not permit estimation of an evolutionary rate. The route by which the male sand flies became infected could not be determined and no vertebrate serosurvey was undertaken; the proposed maintenance of SALV within the vector population therefore remains a hypothesis requiring further validation.

In conclusion, a wild-type SALV strain was successfully isolated for the first time in 64 years from male sand flies in the Aegean region of Türkiye, and the full-length genome sequence was determined. The data indicate that the two strains differ substantially at the nucleotide level while their proteins remain almost identical, and are consistent with maintenance of the virus within the vector population. Given the current concerns about the expansion of sand fly habitats due to climate change, monitoring the dynamics of historically overlooked viruses with unknown pathogenicity, such as the SALV complex, is critical. It is imperative to comprehensively evaluate the risk of this virus emerging as a novel infectious threat through broad vector and host surveillance efforts based on established genomic data.

## Supplementary Information

Below is the link to the electronic supplementary material.

Supplementary Material 1 (DOCX 16.6 KB)

## Data Availability

The genome sequences of SALV strain determined in this study were deposited in the International Nucleotide Sequence Database (DDBJ/EMBL/GenBank). The accession numbers are LC938656 (L segment), LC938657 (M segment), and LC938658 (S segment).
