# Supplementary material for: Genomic analysis of Salehabad virus obtained from the field after a 64-year silence
Source: Arch Virol. 2026 Jul 30;171(8):239. doi: 10.1007/s00705-026-06701-6 (PMC13423903; doi:10.1007/s00705-026-06701-6)
Supplement: Supplementary file 1 — Supplementary Material 1 (DOCX 16.6 KB) [file 705_2026_6701_MOESM1_ESM.docx]

Supplementary Table 1. Viral sequences used in this study.

|  |  | GenBank accession nos. | | |
| --- | --- | --- | --- | --- |
| Virus name | strain | L | M | S |
| Salehabad virus | I-81 | JX472403 | JX472404 | JX472405 |
| Zaba virus | C48 | MG573142 | MG573143 | MG573147 |
| Medjerda Valley virus | T131 | KU255114 | KU255115 | KU297253 |
| Arbia virus | ISS PHL18 | JX472400 | JX472401 | JX472402 |
| Alcube virus | S20 | KR363190 | KR363191 | KR363192 |
| Shable virus | SP109-KE-2019 | PV963161 | PV963159 | PV963157 |
| Adana virus | 195 | KJ939330 | KJ939331 | KJ939332 |
| Bregalaka virus | M31 | MG573144 | MG573145 | MG573146 |
| Grapi virus | Kosovo.SP59 | PV368852 | PV368853 | PV368854 |
| Ponticelli I virus | 194246/2013 | KX388214 | KX388215 | KX388216 |
| Ponticelli II virus | 238134-4/2016 | MG911981 | MG911982 | MG911983 |
| Ponticelli III virus | 195684-2/2016 | MG911984 | MG911985 | MG911986 |
| Arumowot virus | Ar 1286-64 | MF593931 | MF593932 | MF593933 |
| Odrenisrou virus | ArA1131/80 | HM566174 | HM566173 | HM566175 |
| Salanga virus | AnB 904a | KC669549 | KC669550 | KC669551 |
| Sandfly fever Sicilian virus | Izmir 19 | GQ847513 | GQ847512 | GQ847511 |
